# Nicotine Inhibits* Clostridium difficile* Toxin A-Induced Colitis but Not Ileitis in Rats

**DOI:** 10.1155/2016/4705065

**Published:** 2016-01-11

**Authors:** Steven R. Vigna

**Affiliations:** ^1^Departments of Cell Biology & Medicine, Duke University Medical Center, Durham, NC 72210, USA; ^2^Durham VA Medical Center, Durham, NC 27705, USA

## Abstract

Nicotine is protective in ulcerative colitis but not Crohn's disease of the small intestine, but little is known about the effects of nicotine on* Clostridium difficile* toxin A-induced enteritis. Isolated ileal or colonic segments in anesthetized rats were pretreated with nicotine bitartrate or other pharmacological agents before intraluminal injection of toxin A. After 3 hours, the treated segments were removed and inflammation was assessed. Nicotine biphasically inhibited toxin A colitis but not ileitis. Pretreatment with the nicotinic receptor antagonist, hexamethonium, blocked the effects of nicotine. Pretreating the colonic segments with hexamethonium before toxin A administration resulted in more inflammation than seen with toxin A alone, suggesting that a tonic nicotinic anti-inflammatory condition exists in the colon. Nicotine also inhibited toxin A-induced increased colonic concentrations of the TRPV1 (transient receptor potential vanilloid subtype 1) agonist, leukotriene B_4_ (LTB_4_), and release of the proinflammatory neuropeptide, substance P. Pretreatment with nicotine did not protect against direct TRPV1-mediated colitis caused by intraluminal capsaicin. Nicotinic cholinergic receptors tonically protect the colon against inflammation and nicotine inhibits toxin A colitis but not toxin A ileitis in rats in part by inhibition of toxin A-induced activation of TRPV1 by endogenous TRPV1 agonists such as LTB_4_.

## 1. Introduction

*Clostridium difficile* toxin A causes colitis in people and severe acute inflammation in the small intestine and colon of research animals. Little is known about the effects of smoking and nicotine on* C. difficile* colitis. In contrast, smoking is well known to have differential effects in the human inflammatory bowel diseases, ulcerative colitis (UC), and Crohn's disease (CD). It has been shown that current smoking protects against UC [1, 2] but not CD and is beneficial after the onset of UC. In addition, smoking cessation worsens the course of UC in people. Ulcerative colitis has a more benign disease course in smokers than in nonsmokers in terms of flare-ups, hospitalization rates, the need for oral steroids, and colectomy rates [1]. Smoking also appears to be protective in indeterminate colitis as well as in UC [1]. On the other hand, smoking is a risk factor for CD and has been shown to be a neutral or an aggravating factor in this disease [3]. Interestingly, smoking might determine disease location in CD because most studies report a higher prevalence of ileal disease and a lower prevalence of colonic involvement in smokers with CD [1]. This concept is supported by the results from a prospective study that found that patients with CD who smoke are less likely to have colonic involvement, suggesting that smoking protects the colon from inflammation in CD as it does in UC [4]. These results and others have led to speculation that the protective effects of smoking in the gut are not disease specific but may instead be colon specific [5].

Nicotine has been shown to be the active protective component in tobacco smoke because nicotine transdermal patches or nicotine enemas protect the colon in UC [6, 7]. It has been reported that smokers have higher rates of* C. difficile* infections than nonsmokers in Americans older than 50 [8] but there are few mechanistic studies of the effects of nicotine on toxin A-induced acute inflammation of the intestines. The goal of the present studies was to determine the effects of nicotine on* C. difficile* toxin A-induced ileitis and colitis in rats.

## 2. Materials and Methods

### 2.1. Materials

(−)-Nicotine hydrogen tartrate, hexamethonium, and capsaicin were purchased from Sigma (St. Louis, MO).* Clostridium difficile* toxin A was purchased from TeckLab, Inc. (Blacksburg, VA). LTB_4_ enzyme immunoassay (EIA) kits were purchased from Cayman Chemical (Ann Arbor, MI).

### 2.2. Surgery

Isolated colonic segments were constructed in anesthetized male Sprague-Dawley rats (150–175 g) as previously described for construction of ileal segments [9, 10]. Isolated colonic segments 5 cm in length were constructed distal to the caecum by ligation with silk sutures.

### 2.3. Drug Administration

Toxin A was administered at a dose of 5 *μ*g in 400 *μ*L (or 200 *μ*L when given after other drugs) of PBS into the lumen of the isolated intestinal segments.

(−)-Nicotine hydrogen tartrate (“nicotine”) was dissolved in 200 *μ*L (or 100 *μ*L when given after other drugs) of PBS and administered at doses of 2 ng–20 *μ*g into the lumen of isolated intestinal segments 30 min prior to toxin A or capsaicin injection. Hexamethonium was dissolved in 200 *μ*L (or 100 *μ*L when given before nicotine) of PBS and administered at a dose of 5 *μ*g into the lumen of isolated intestinal segments 30 min prior to other drugs. Capsaicin was administered at a dose of 4 mg in 400 *μ*L (or 200 *μ*L when given after other drugs) of 25% ethanol in saline. All intraluminal injections were made using a 27 ga syringe needle. Control rats were prepared identically and their isolated colonic segments were injected with the appropriate vehicle solutions. These studies were approved by the Duke University and Durham VA Institutional Animal Care and Use Committees.

### 2.4. Luminal Fluid Accumulation

Luminal fluid accumulation was measured gravimetrically. After 3 hours of treatment, the isolated ileal segments were removed and weighed and their lengths were measured. Luminal fluid accumulation is expressed as mg wet weight per cm length.

### 2.5. Myeloperoxidase Activity

Myeloperoxidase (MPO) activity was measured as described previously [11]. Briefly, pieces of control and treated ileal segments were homogenized in 0.5% hexadecyltrimethylammonium bromide in 50 mM KH_2_PO_4_ (pH 6), frozen/thawed three times, and centrifuged at 4°C for 2 minutes, and then the absorbance of each supernatant was read at 460 nm at 0, 30, and 60 seconds after the addition of 2.9 mL of* o*-dianisidine dihydrochloride to 0.1 mL supernatant. The maximal change in absorbance per minute was used to calculate the units of MPO activity based on the molar absorbency index of oxidized* o*-dianisidine of 1.13 × 10^4^ M^−1^ cm^−1^. The results are expressed as MPO units of activity per gram of tissue wet weight.

### 2.6. LTB_4_ Measurement

Intestinal LTB_4_ levels were measured by LTB_4_ enzyme immunoassay (EIA) kits purchased from Cayman Chemical (Ann Arbor, MI) as previously described [12]. Briefly, samples of ileum and ileal luminal fluid were collected after various treatments in 5 volumes of ice-cold 0.1 M phosphate buffer, pH 7.4, containing 1 mM EDTA and 10 *μ*M indomethacin and homogenized for 15 sec on ice using a Tekmar Tissumizer (Tekmar, Cincinnati, OH) at a 50% power setting. Before homogenization, 10,000 cpm of ^3^H-LTB_4_ (120–240 Ci/mmol, Perkin-Elmer Life Sciences, Boston, MA) was added to the buffer for later assessment of LTB_4_ recovery. After homogenization, 2 volumes of ice-cold ethanol were added to each extract and the extracts were then incubated on ice for 5 min to precipitate proteins. After centrifugation at 3000 ×g_max_ to remove the precipitated proteins, the ethanol in the supernatants was removed by vacuum centrifugation. The pH of the extracts was adjusted to ~4.0 by addition of 1 M sodium acetate (pH 4.0). The resulting precipitate was removed by centrifugation and the supernatant was loaded onto C-18 solid phase extraction cartridges (Cayman Chemical, Ann Arbor, MI) previously washed with methanol and distilled water, washed with distilled water followed by hexane, and then eluted at unit gravity with 5 mL of 99% ethyl acetate: 1% methanol. The samples were then evaporated to dryness by vacuum centrifugation, reconstituted in LTB_4_ EIA buffer, and assayed according to the instructions of the kit manufacturer. Because toxin A caused portions of the ileal mucosa to slough off into the intestinal lumen, luminal contents were collected by syringe from toxin A-treated ileal segments, assayed for LTB_4_ content just as for ileal tissue, and the LTB_4_ contents of the ileal tissue and corresponding luminal contents were added together for these samples. Because it seemed inappropriate to express the results as LTB_4_ concentrations per unit wet weight, therefore, the results are expressed as LTB_4_ concentrations per cm of ileal length.

### 2.7. Substance P Release

Substance P release was assessed by analysis of NK-1R endocytosis as described previously [13, 14] with modifications. Briefly, pieces of ileal segments taken from control, toxin A-treated, and capsazepine pretreated/toxin A-treated rats were fixed in freshly depolymerized 4% paraformaldehyde overnight at 4°C. The tissue was then washed and embedded in Tissue Tek O.C.T. compound (Sakura, Torrance, CA), frozen, sectioned at 20 *μ*m, and mounted on Superfrost/Plus glass slides (Fisher, Pittsburgh, PA). After washing, the slides were stained using a rabbit antiserum (#11886-5) specific for the C-terminal 15 amino acids of the rat NK-1R at a dilution of 1 : 3000 [15]. This was followed by incubation in a cyanine 3-conjugated donkey anti-rabbit IgG secondary antibody (Jackson ImmunoResearch, West Grove, PA) at a dilution of 1 : 600. The stained sections were analyzed using a Zeiss LSM-510 META inverted krypton-argon confocal laser scanning system coupled to a Zeiss Axiovert 200 MOT microscope. Images of 512 × 512 pixels were obtained and processed using Adobe Photoshop. Quantification of NK-1R endocytosis was achieved by analyzing 20 NK-1R-immunoreactive (NK-1R-ir) myenteric plexus neuronal cell bodies per rat and determining the number of these cells containing more than 10 NK-1R-ir endosomes. Cytoplasmic NK-1R-ir endosomes were distinguished from NK-1R-ir plasma membranes or plasma membrane-associated endosomes by ensuring that the nucleus of the neurons was in the same optical section as the NK-1R-ir endosomes.

### 2.8. Statistical Analysis

Results are expressed as mean ± SEM (*N* = 5–7). Mean differences among groups were assessed by one-way ANOVA and the Tukey-Kramer multiple comparisons posttest using GraphPad Prism for Windows (GraphPad Software, San Diego, CA). In some cases when Bartlett's test revealed that the differences among the standard deviations of the various groups were significant, the data were analyzed by the Kruskal-Wallis nonparametric ANOVA followed by Dunn's multiple comparisons test. *P* values < 0.05 were considered significant. The numbers of rats per group are given in the figure legends.

## 3. Results

**Figure 1:**
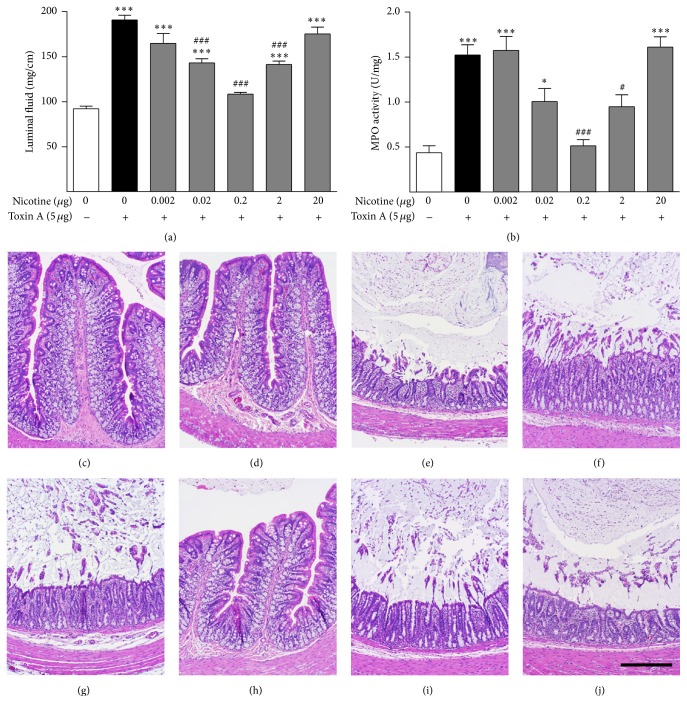
Dose-response effects of nicotine on toxin A-induced (5 *μ*g) colonic luminal fluid accumulation (a), MPO content (b), and histopathology (c–j). Toxin A strongly and significantly stimulates colonic luminal fluid accumulation and MPO activity. Nicotine has a biphasic effect on these actions of toxin A. At low doses, nicotine dose-dependently inhibited these effects of toxin A. At higher doses, nicotine inhibited toxin A colitis progressively less but never increased inflammation beyond the effect of toxin A alone. ^*∗∗∗*^ *P* < 0.001 versus control; ^###^ *P* < 0.001 versus toxin A; *N* = 6. (c) Control section illustrating the normal histology of the rat colon. (d) Nicotine (0.2 *μ*g) alone has no effect on colonic histology. (e) Toxin A (5 *μ*g) causes extensive inflammatory damage to the colon including loss of mucosal folding, distortion of crypts, surface ulceration, and influx of inflammatory cells including neutrophils. (f) Toxin A (5 *μ*g) after pretreatment with 0.002 *μ*g nicotine. This dose of nicotine had little effect on toxin A colitis. (g) Toxin A (5 *μ*g) after pretreatment with 0.02 *μ*g nicotine. This dose of nicotine had little effect on toxin A colitis. (h) Toxin A (5 *μ*g) after pretreatment with 0.2 *μ*g nicotine. This dose of nicotine appeared to protect almost completely against toxin A colitis. (i) Toxin A (5 *μ*g) after pretreatment with 2 *μ*g nicotine. This dose of nicotine had little effect on toxin A colitis. (j) Toxin A (5 *μ*g) after pretreatment with 20 *μ*g nicotine. This dose of nicotine had little effect on toxin A colitis. These results agree well with the effects of nicotine on toxin A-induced luminal fluid accumulation and MPO activity shown in (a) and (b). Line bar = 100 *μ*m.

Intraluminal administration of toxin A to the rat colon for 3 hours causes intense inflammation including increased intraluminal fluid accumulation, tissue MPO content, and histopathology (Figure 1). The histological effects included flattening of mucosal folding, surface ulceration, disruption of normal crypt architecture, and extensive infiltration of inflammatory cells most prominently including neutrophils. Pretreatment for 30 minutes with intraluminal nicotine in the ng range resulted in a dose-dependent inhibition of the toxin A-induced luminal fluid accumulation, MPO activity, and histopathology. However, the effects of nicotine on these indices of intestinal inflammation were biphasic because a higher nicotine dose of 20 *μ*g had no effect.

**Figure 2:**
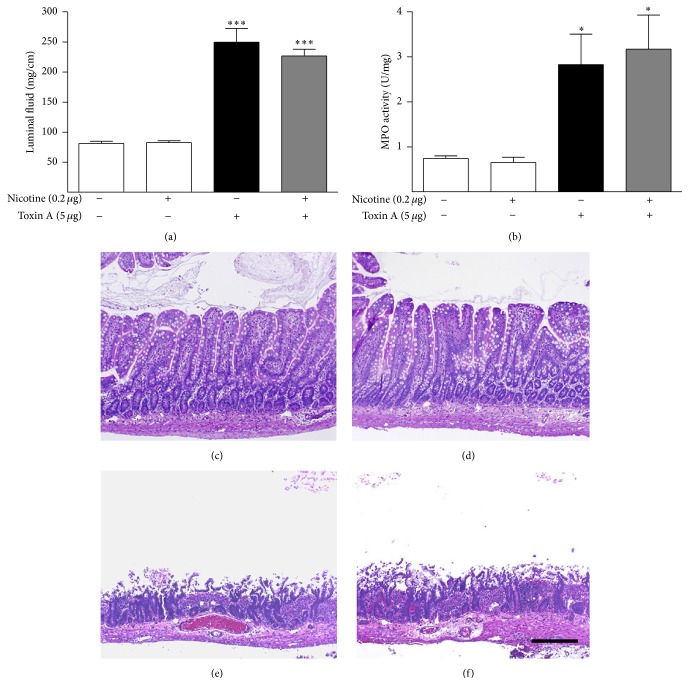
Lack of effect of nicotine on toxin A-induced luminal fluid accumulation (a), MPO content (b), and histopathology in the ileum (c–f). Nicotine pretreatment had no effect on toxin A-stimulated ileal luminal fluid accumulation (a) or MPO content (b). ^*∗*^ *P* < 0.05 versus control; ^*∗∗∗*^ *P* < 0.001 versus control; *N* = 6. (c) Control section illustrating the normal histology of the rat ileum. (d) Nicotine (0.2 *μ*g) alone had no effect on ileal histology. (e) Toxin A (5 *μ*g) caused extensive inflammatory damage to the ileum including complete loss of villi, crypt distortion, surface ulceration, and influx of neutrophils. (f) Toxin A (5 *μ*g) after pretreatment with 0.2 *μ*g nicotine. This dose of nicotine was maximally effective against toxin A in the colon but did not protect the ileum. Line bar = 100 *μ*m.

Interestingly, pretreating the ileum with the dose of nicotine found to be most protective in the colon did not inhibit toxin A-induced inflammation in this segment of the small intestine (Figure 2). Higher and lower doses of nicotine were also ineffective in the ileum (data not shown).

**Figure 3:**
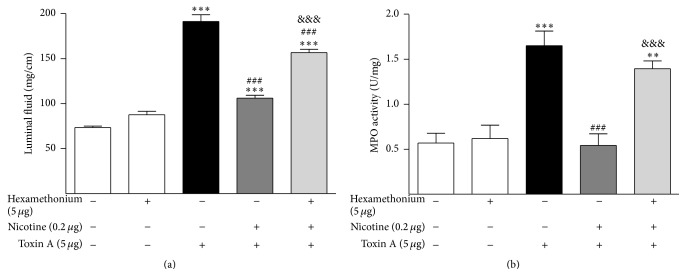
The anti-inflammatory effect of nicotine on the rat colon is mediated by nicotinic receptors because the specific nicotinic receptor antagonist, hexamethonium, significantly inhibited the protective effects of nicotine on toxin A-induced luminal fluid accumulation (a) and MPO content (b) in the colon. ^*∗∗*^ *P* < 0.01 versus control; ^*∗∗∗*^ *P* < 0.001 versus control; ^###^ *P* < 0.001 versus toxin A; ^&&&^ *P* < 0.001 versus toxin A + nicotine; *N* = 6.

In order to determine if the protective effect of nicotine in the colon is mediated by the cholinergic nicotinic receptor rather than by some other nonspecific effect, the colon was pretreated with the specific nicotinic receptor antagonist, hexamethonium. Hexamethonium at the dose of 5 *μ*g abolished the protective effects of nicotine against toxin A-induced luminal fluid accumulation and MPO content (Figure 3).

**Figure 4:**
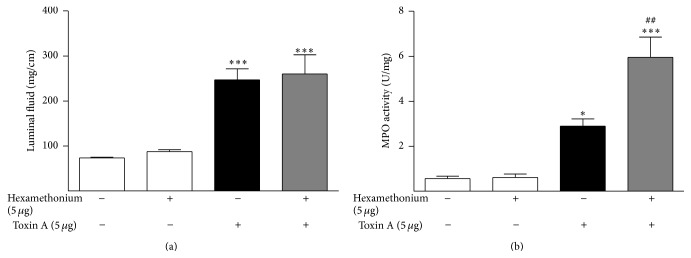
Effects of pretreatment with hexamethonium (5 *μ*g) on toxin A-induced (5 *μ*g) colonic luminal fluid accumulation (a) and MPO content (b). Administration of hexamethonium alone had no effect on colonic luminal fluid accumulation, MPO content, or toxin A-stimulated luminal fluid accumulation but significantly increased toxin A-stimulated MPO content. ^*∗*^ *P* < 0.05 versus control; ^*∗∗∗*^ *P* < 0.001 versus control; ^##^ *P* < 0.01 versus toxin A alone; *N* = 6.

The effect of pretreating the colon with hexamethonium (in the absence of nicotine treatment) on toxin A colitis was examined next. Hexamethonium has previously been shown to worsen DNBS-induced colitis in rats [16], suggesting that there is a tonic nicotine receptor-mediated protection of the colon against inflammation. Hexamethonium alone had no effect on colonic indices of inflammation but significantly exacerbated toxin A-induced increased colonic MPO content (there was no significant effect on toxin A-induced luminal fluid accumulation, Figure 4).

**Figure 5:**
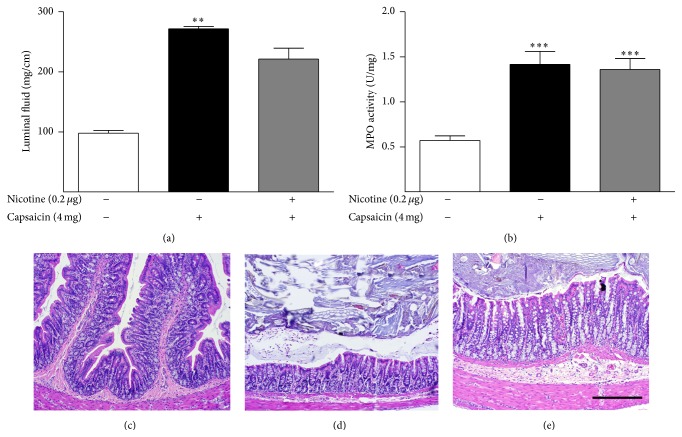
Effects of pretreatment with nicotine (0.2 *μ*g) on capsaicin-induced (4 mg) luminal fluid accumulation (a) and MPO content (b) in the colon. Capsaicin is a direct TRPV1 agonist. Nicotine pretreatment had no effect on capsaicin-stimulated colitis in this model, unlike the effect of nicotine on toxin A-stimulated colitis as shown. ^*∗∗*^ *P* < 0.01 versus control; ^*∗∗∗*^ *P* < 0.001 versus control; *N* = 5. (c) Control section illustrating the normal histology of the rat colon; (d) capsaicin (4 mg) causes extensive inflammatory damage to the colon including loss of mucosal folding, crypt distortion, surface ulceration, and influx of neutrophils; (e) capsaicin (4 mg) after pretreatment with 0.2 *μ*g nicotine. This dose of nicotine was maximally effective against toxin A in the colon but did not protect against capsaicin. Line bar = 100 *μ*m.

As a first step in testing the hypothesis that nicotine acts to protect against colitis by inhibiting the generation of a proinflammatory TRPV1 agonist in the colon, the effect of pretreating the colon with nicotine before administering capsaicin, a direct TRPV1 agonist that has previously been shown to be highly inflammatory in the rat ileum [13], was tested. Intraluminal capsaicin was also highly inflammatory in the rat colon (Figure 5). Unlike the results using toxin A as the inflammatory agent, nicotine pretreatment did not protect against capsaicin-induced colitis. Since capsaicin is a direct TRPV1 agonist, this result suggests that if toxin A causes an increase in an endogenous TRPV1 agonist that subsequently mediates inflammation, then nicotine may act by inhibiting the generation of this agonist in the colon.

**Figure 6:**
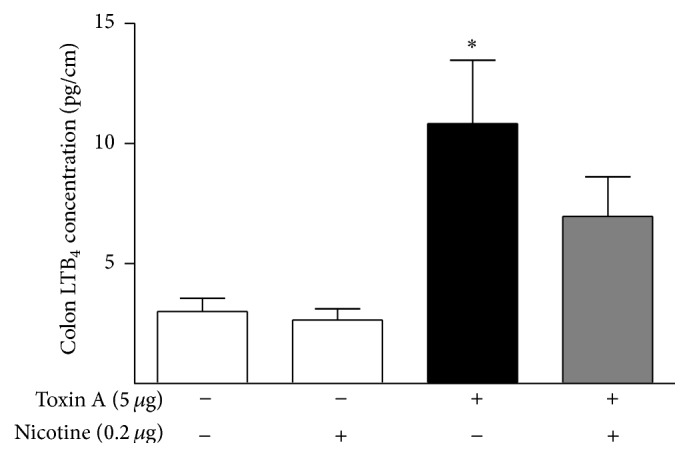
The effects of toxin A with and without pretreatment with nicotine on colonic LTB_4_ content. Toxin A significantly stimulated colonic LTB_4_ content and this effect was inhibited by pretreatment with nicotine but was not statistically significant (*P* = 0.23). ^*∗∗*^ *P* < 0.05 versus toxin A−/nicotine−; *N* = 9.

It has previously been shown that leukotriene B_4_ (LTB_4_) levels are increased in the ileum in response to intraluminal toxin A [12] and LTB_4_ is a well-characterized endogenous TRPV1 agonist [17]. Therefore, endogenous colonic LTB_4_ concentrations were measured in the colon in response to toxin A with and without nicotine pretreatment. Toxin A stimulated a significant increase in colonic tissue LTB_4_ content (Figure 6). Nicotine pretreatment inhibited the effect of toxin A on tissue LTB_4_ levels.

**Figure 7:**
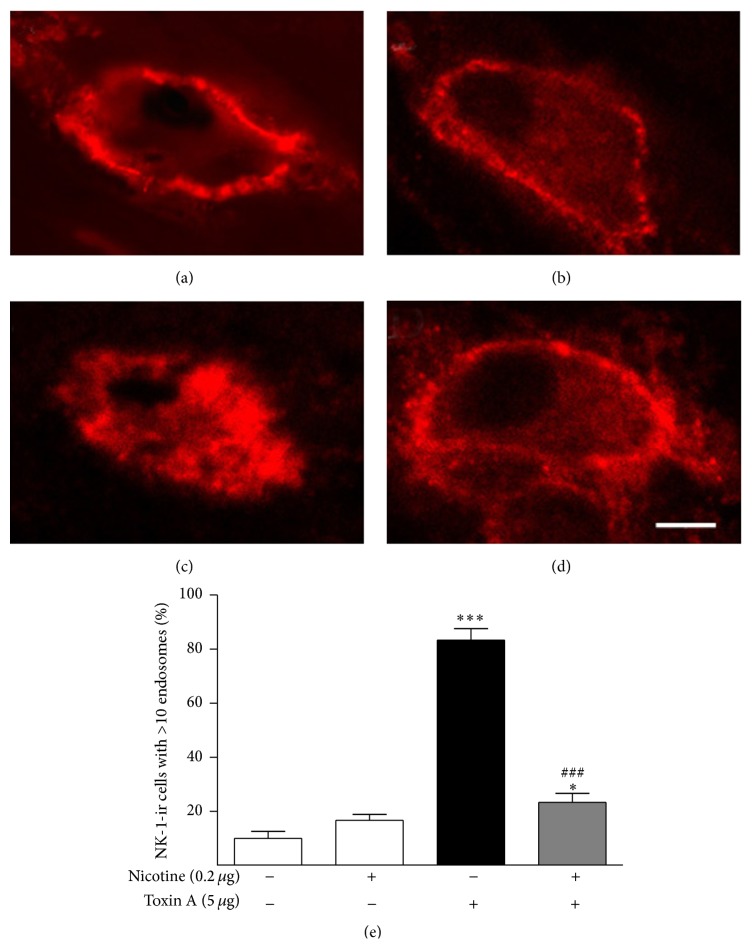
Effect of nicotine pretreatment on toxin A-induced release of endogenous substance P (SP) in the rat colon. (a) Confocal image of neurokinin-1 (NK-1) receptor distribution on a myenteric plexus neuronal ganglionic cell body in a control rat treated with vehicle. The NK-1 receptor is localized on the plasma membrane. (b) Confocal image of neurokinin-1 (NK-1) receptor distribution on a myenteric plexus neuronal ganglionic cell body in a control rat treated with nicotine (0.2 *μ*g). Nicotine alone has no effect on SP-induced NK-1 receptor endocytosis. (c) Confocal image of neurokinin-1 (NK-1) receptor distribution on a myenteric plexus neuronal ganglionic cell body in a rat treated with toxin A (5 *μ*g). Toxin A causes endogenous SP release seen here as NK-1 receptor endocytosis. (d) Confocal image of neurokinin-1 (NK-1) receptor distribution on a myenteric plexus neuronal ganglionic cell body in a rat pretreated with nicotine (0.2 *μ*g) before toxin A (5 *μ*g). Nicotine prevented toxin A-induced SP release in colon. Line bar = 100 *μ*m. (e) Quantitation of the effects of toxin A and nicotine on SP release in the rat colon. ^*∗*^ *P* < 0.05 versus control; ^*∗∗∗*^ *P* < 0.001 versus control; ^###^ *P* < 0.001 versus toxin A; *N* = 6.

More evidence that nicotine acts by inhibiting the ability of toxin A to stimulate the production of an endogenous TRPV1 agonist in the colonic mucosa was provided by the demonstration that nicotine inhibits toxin A-stimulated substance P (SP) release in the colon (Figure 7). Intraluminal toxin A caused a highly significant increase in the endocytosis of the substance P neurokinin-1 receptor (NK-1R), an index of endogenous SP release [18–25].

## 4. Discussion

The main findings in this study are that nicotinic cholinergic receptors protect the colon against* C. difficile* toxin A-induced inflammation tonically and, in addition, that pretreatment of the colon with nicotine further inhibits toxin A-induced colitis. In contrast, nicotine had no protective effect on toxin A-induced ileitis, even though the inflammatory responses of both the colon and the ileum to toxin A are similar. This pattern closely resembles that previously observed in various other animal models of colitis and in the human inflammatory bowel diseases, UC and CD. These observations suggest that there may be similarities in the colonic responses to acute versus chronic inflammation.

Administration of the specific nicotinic receptor antagonist, hexamethonium, by itself had no effect on colonic indices of inflammation but significantly exacerbated toxin A-induced increased MPO activity. The findings that nicotine protected the colon but not the ileum are interesting in light of the results of Sörensson et al. who reported that hexamethonium attenuated the effects of toxin A on several indices of inflammation in rat jejunal-ileal segments [26], further supporting the differential effects of nicotinic cholinergic receptors in the colon versus the small intestine.

The effects of smoking and nicotine on intestinal inflammation have also been examined in several other animal models of intestinal inflammation. In the dinitrobenzenesulfonic acid (DNBS) model of colitis in rats, nicotine had a biphasic effect, improving inflammation at low doses and enhancing it at higher doses [16]. Similar findings were reported by another group who showed in addition that passive cigarette smoke inhibited DNBS-stimulated colonic leukotriene B_4_ (LTB_4_) and TNF*α* levels [27]. In the trinitrobenzenesulfonic acid (TNBS) model of colitis in rats, nicotine also had a dose-dependent biphasic effect, significantly reducing damage at low nicotine doses and having little effect at high nicotine doses [28]. Similar results were noted in another TNBS study in rats in which it was also observed that low doses of nicotine that inhibited inflammation also reduced colonic tissue levels of LTB_4_ and tumor necrosis factor *α* (TNF*α*) [29]. The conclusion that nicotine inhibits colonic TNF*α* levels was supported in a study of the mouse colon [30]. In contrast, two groups have reported that cigarette smoke aggravates DNBS or TNBS colitis in rats [31, 32], but this may possibly be explained by the biphasic dose-response effect of nicotine observed earlier in which low doses are protective and high doses are not.

In the iodoacetamide model of enteritis in rats, nicotine was found to be protective against iodoacetamide-induced colitis but not jejunitis [33]. Iodoacetamide was judged to cause a form of intestinal inflammation more closely resembling human CD than UC, suggesting that perhaps the regional differences in the effects of nicotine on the intestine may provide a possible explanation for the differential effects of smoking in human UC versus CD. Similar findings were reported in another animal model also judged to more closely resemble human CD than UC, the interleukin-10 (IL-10) knockout mouse model. Genetic ablation of IL-10 results in both colitis and jejunitis in these animals. Nicotine treatment resulted in significant protection of the colon but the jejunum was more severely damaged [34]. The concept that nicotine has different effects on the small intestine and colon was further supported by studies showing that nicotine has different regional effects on small bowel and colonic mucosal cytokine levels [35].

The mechanism by which nicotinic cholinergic stimulation protects the colon against toxin A damage is unknown, but recent studies have demonstrated that a powerful cholinergic anti-inflammatory reflex exists in which neural detection of inflammatory agents reflexly stimulates vagal release of acetylcholine peripherally to stimulate nicotinic receptors that inhibit macrophage and dendritic cell immune responses [36–38]. This cholinergic anti-inflammatory pathway has been demonstrated to be active in such diverse experimental inflammatory models as sepsis [36], ischemic reperfusion [39], hemorrhage [40], and postoperative ileus [41]. This reflex may be part of the effects of nicotine on toxin A-induced colitis described here but other mechanisms may also be involved. It has been shown previously that intraluminal toxin A stimulates primary sensory neurons in the intestinal mucosa via activation of the transient receptor potential vanilloid subtype 1 (TRPV1) receptor to release substance P (SP) ultimately resulting in intestinal inflammation [13]. However, the mechanism by which intraluminal toxin A activates TRPV1 is unknown. The first step in toxin A action is binding to mucosal epithelial cells at their apical microvilli [42]. It has been shown that toxin A binds to villous but not crypt epithelial cells in the small intestine and also does not bind to goblet cells [43]. Thus toxin A exerts its effects at the level of the mucosal epithelium and does not penetrate deeper into the intestinal mucosa. This is consistent with the observations that when toxin A binds to cells it is rapidly internalized and causes glucosylation of Rho proteins, decreased protein synthesis, and finally cellular necrosis [44]. These observations led to the hypothesis that toxin A stimulates production of an endogenous TRPV1 agonist in the mucosal epithelium which in turn activates TRPV1-expressing sensory neurons in the adjacent lamina propria. There is evidence that LTB_4_ may be the endogenous TRPV1 agonist stimulated by toxin A in the rat intestine [12]. The current results provide three lines of evidence supporting the concept that a neurogenic mechanism involving TRPV1 activation plays a role in nicotinic inhibition of colitis. First, when inflammation was induced by capsaicin instead of toxin A, nicotine had no effect. Capsaicin is a direct agonist of TRPV1, unlike toxin A which causes the release of an endogenous TRPV1 agonist [12–14]. Thus, the lack of inhibition of capsaicin-induced inflammation by nicotine demonstrates that nicotine is acting prior to stimulation of TRPV1 in the inflammatory cascade. Second, toxin A stimulated the generation of a potent endogenous TRPV1 agonist in the colon, LTB_4_, and nicotine inhibited this effect. Third, toxin A also stimulated local release of the TRPV1-dependent proinflammatory neuropeptide, substance P, in the colon and nicotine also inhibited this response to the toxin. Taken together, these results support the concept that one of the mechanisms by which colonic nicotinic receptor activation resists inflammation is by inhibition of the generation of endogenous TRPV1 agonists such as LTB_4_.

In contrast to the findings here in rats, it has been reported that Americans older than 50 who smoke have higher rates of* C. difficile* infections than nonsmokers [8]. However, this was a retrospective study in which the dosage of nicotine intake was unknown. In addition, since* Clostridium* species are known to be present in cigarettes [45], smoking may be an oral source of spores into the gastrointestinal tract in smokers. Furthermore, it is possible that smokers are more likely than nonsmokers to receive antibiotics, which are strongly associated with* C. difficile* infection [42]. In light of these considerations, it may be useful to examine more closely the effects of smoking and nicotine in human* C. difficile* infections.

## 5. Conclusions

Nicotinic cholinergic receptors tonically protect the colon against inflammation and nicotine inhibits toxin A colitis but not ileitis in rats in part by inhibition of toxin A-induced activation of TRPV1 by endogenous TRPV1 agonists such as LTB_4_.
